# Bronchial Epithelial Cells on the Front Line to Fight Lung Infection-Causing *Aspergillus fumigatus*

**DOI:** 10.3389/fimmu.2020.01041

**Published:** 2020-05-22

**Authors:** Jeanne Bigot, Loïc Guillot, Juliette Guitard, Manon Ruffin, Harriet Corvol, Viviane Balloy, Christophe Hennequin

**Affiliations:** ^1^Sorbonne Université, Inserm, Centre de Recherche Saint-Antoine, CRSA, AP-HP, Hôpital Saint-Antoine, Service de Parasitologie-Mycologie, Paris, France; ^2^Sorbonne Université, Inserm, Centre de Recherche Saint-Antoine, Paris, France; ^3^Sorbonne Université, Inserm, Centre de Recherche Saint-Antoine, CRSA, AP-HP, Hôpital Trousseau, Service de Pneumologie Pédiatrique, Paris, France

**Keywords:** bronchial epithelial cells, *Aspergillus fumigatus*, innate immunity, lung infection, mucociliary machinery

## Abstract

*Aspergillus fumigatus* is an environmental filamentous fungus that can be pathogenic for humans, wherein it is responsible for a large variety of clinical forms ranging from allergic diseases to life-threatening disseminated infections. The contamination occurs by inhalation of conidia present in the air, and the first encounter of this fungus in the human host is most likely with the bronchial epithelial cells. Although alveolar macrophages have been widely studied in the *Aspergillus*–lung interaction, increasing evidence suggests that bronchial epithelium plays a key role in responding to the fungus. This review focuses on the innate immune response of the bronchial epithelial cells against *A. fumigatus*, the predominant pathogenic species. We have also detailed the molecular interactants and the effects of the different modes of interaction between these cells and the fungus.

## Introduction

*Aspergillus* spp. are saprophytic filamentous fungi capable of colonizing different ecological habitats. They are usually isolated from soils, decaying organic matters, and plants but are also present in the air and indoor environments (1). *Aspergillus* spores, or so-called conidia, represent the disseminating form of the fungus that spreads through the air. These conidia are produced through asexual reproduction by differentiated fungal cells called phialides, which are themselves carried on a conidiophore. Conidia remain “dormant” or metabolically inactive until they encounter favorable environmental conditions. In this case, the conidia swell, germinate to produce hyphae that grow into a mycelium that harbor conidiophores, and then form conidia (2).

Aspergillus genus encompasses several hundred of species (3). *Aspergillus fumigatus* is by far the most frequent pathogenic species, responsible for about 90% of the cases of *Aspergillus* diseases, followed by *Aspergillus flavus, Aspergillus niger, Aspergillus terreus*, and *Aspergillus nidulans* (3, 4). Indeed, *A. fumigatus* is the predominant fungal species isolated from the ambient air able to grow at 37°C, the human body temperature.

Humans inhale around a few hundred conidia daily (5). Due to their small size (2- to 3-μm diameter) they can reach the lower respiratory tract (4) but, in most of cases, this phenomenon does not lead to any symptoms thanks to their evacuation by the mucociliary machinery of the tracheobronchial epithelium. However, depending on the immune status of the host, this contamination can be followed by a wide spectrum of manifestations (1). Concisely, immunocompromised patients are at risk for invasive infection, so-called invasive pulmonary aspergillosis (IPA) and patients with pre-formed lung cavity (typically following previous pulmonary tuberculosis) are prone to chronic pulmonary aspergillosis, of which aspergilloma is one of the main presentations. Finally, patients with altered mucociliary clearance, such as cystic fibrosis (CF) patients, may be colonized which can turn, in patients with exacerbated immune response, into allergic bronchopulmonary aspergillosis (ABPA).

The essential role of neutrophils and monocytes in anti-*Aspergillus* immunity has been emphasized by the high rate of incidence of *Aspergillus* invasive infection in patients with quantitative (neutropenia) or qualitative (corticosteroid therapy, chronic granulomatous disease) deficiency of these cells (6–8). However, the role of the bronchial epithelium should not be underestimated as it represents the first physical and biological barrier preventing fungal implantation.

While studies looking at the interactions between *Aspergillus* and leukocytes (alveolar macrophages and recruited neutrophils) are numerous (6, 9–11), data on the role of bronchial epithelial cells (BECs) in anti-*Aspergillus* defense are still limited. Yet, BECs seem to play a crucial role in the innate immune response against *Aspergillus* particularly in preventing the bronchial colonization. The high prevalence of *Aspergillus* bronchial colonization in patients suffering from CF (12, 13), a disease characterized by the thickening of the bronchial mucus, highlights this phenomenon. Bronchial *Aspergillus* colonization, whose role in the subsequent development of IPA is still debated, may have deleterious consequences as it is the starting point of *Aspergillus* bronchitis and immuno-allergic forms (14, 15). In CF patients, while remaining superficial, bronchial colonization is associated with the occurrence of bronchial exacerbations, a decline in lung function, and ABPA with a prevalence ranging between 1 and 15% (16). Fungal sensitization to *Aspergillus* antigens may also occur in allergic patients (17) but the role of the bronchial epithelium in these diseases won't be analyzed in this review.

Thanks to experimental studies, there is increasing knowledge on the interactions between the different morphotypes of *A. fumigatus* and BECs. This review aims to decipher these interactions at the molecular level and their effect on anti-*Aspergillus* immunity.

## Study Models of the Interaction Between *Aspergillus fumigatus* and Bronchial Epithelial Cells

The respiratory tract is lined by epithelial cells whose types vary according to the anatomic structure of the airways. Trachea, bronchi, and bronchioles are lined by the pseudostratified epithelium, while type I and II pneumocytes constitute the alveolar epithelium. At the bronchial level, the pseudostratified epithelium is mostly composed of ciliated, secretory, and basal cells from which the first two derive.

To understand the interactions between *Aspergillus* and BECs, different cell lines (immortalized or tumor) have been commonly used. Among the most popular bronchial cell lines used and commercially available, we can cite BEAS-2B and 16HBE, both isolated from normal human bronchial epithelium and secondarily immortalized through transfection of a replication-defective SV40 plasmid (18, 19). NCI-H292 cells derive from a lymph node metastasis sample of a pulmonary mucoepidermoid carcinoma. But other respiratory cell lines are occasionally used in some studies. All these cell lines have major advantages such as easy to maintain (cultured in simple and inexpensive culture media), capable of growing at high densities, and exhibiting an extended life span (20). However, these cells represent only one donor, and many cellular processes are deregulated due to immortalization. Refinement of the model consists of the use of commercially available primary bronchial cells that are free from any genetic modification and whose physiological functions are intact. However, before they are used, those cells must undergo antibiotic, antifungal, and growth factor treatment. Usually, these cells have a limited life span with limited proliferation capacity and are more difficult to culture than cell lines requiring more complex, specialized, and expensive cell culture media. Irrespective of the cell type, cells are usually cultured under submerged conditions, i.e., in flat-bottom plastic wells filled with culture medium, that hamper cells to differentiate. To better mimic physiological conditions, air-liquid interface cell culture (ALI) systems have been developed (21). In this case, primary BECs and also some cell lines such as 16HBE, differentiate until they develop the mucociliary phenotype characteristic of a pseudostratified epithelium and express mucins (20). Basal surface is therefore in contact with the liquid medium and the apical part of the cellular layer is exposed to the air. Obtaining this type of differentiated epithelium is time-consuming and requires specific technical skills but such cell culture systems mimic the required *in vivo* conditions in the best way.

Different approaches can be used to mimic an *Aspergillus* infection. *Aspergillus*, mostly in the form of dormant conidia can be inoculated in cell culture supernatants and then recovered after defined incubation intervals to measure the parameters of interest (such as cytokine level, cytotoxicity, etc.). In these conditions, *A. fumigatus* hyphae are usually obtained after 15 h of incubation. Killed (UV or irradiated for example) resting, swollen conidia and hyphae have also been used as inoculum. Differences between experimental protocols, especially the use of different multiplicity of infection, likely far from reality, could explain some discrepancies in the results obtained in different studies.

In addition to *in vitro* models, *in vivo* models of *Aspergillus* infection have already been used. Mice, rats or rabbits are the animals the most commonly used. They are immunosuppressed or not, and infected with *A. fumigatus* through inhalation of conidia administered either intranasal or *via* intratracheal route. In addition to the measurements of mortality rate and/or fungal load in the lungs, more precise descriptions of the immune response have also been reported, looking at the immune cells recruitment or inflammatory response (22). *In vivo* models have the considerable advantage of most closely imitate lung infection and immunity as a whole, however, there is a paucity of *in vivo* models (conditional and inducible transgenic mice targeting bronchial/airway epithelial cells) allowing the study of BECs against *Aspergillus* challenge specifically.

## Anti-*Aspergillus* Physicochemical Activity Exhibited by the Bronchial Epithelium

Inhaled conidia first face the physical barriers of the upper airways that include the mouth, nose, larynx, and pharynx. Mucociliary clearance from the nasal walls and mechanical defenses such as coughing and sneezing help eliminate most of the inhaled particles. If the conidia pass these first barriers, they then arrive in the lower airways consisting of the trachea that divided into two-stem bronchial tubes, which in turn are subdivided into several smaller bronchial tubes, followed by bronchioles that end with the alveoli. The bronchial epithelium participates in the clearance of inhaled conidia to prevent their germination and growth locally. Secretory cells, including serous and goblet cells, together with submucosal glands, participate in the formation of mucus, which protects the epithelium from the inhaled particles. Basically, the mucus traps the inhaled particles, which are then actively transported by the beating of the cilia to the oropharynx where they are swallowed or expectorated. Under healthy conditions, mucus is composed of 97% water and 3% of mucins, non-mucin proteins, salts, lipids, and cellular debris (23). Mucins, namely MUC5AC and MUC5B, are the major macromolecular constituents of the mucus. They are large glycoproteins with serine-/threonine-rich domains linked by their hydroxyl side groups to sugar chains forming a polymeric gel that ensure the properties of the mucus (24). Ciliated cells also play a fundamental role in the elimination of particles engulfed in the mucus because they mechanize the movement of the mucus blanket (25). The role of these physicochemical barriers associated with the bronchial epithelium can be better understood in patients suffering from CF or primary ciliary dyskinesia (PCD) in whom mucus properties and/or ciliary beating are impaired. Hence, CF patients are frequently colonized by *A. fumigatus* (14), and a similar trend has been noticed in patients with PCD (26). This machinery can also be altered by pathogens. Indeed, mycotoxins secreted by *A. fumigatus*, damage epithelial cells and inhibit ciliary beating (27). Among those toxins, gliotoxin has been extensively studied because it is the most abundant one produced by *A. fumigatus* and it exhibits immunosuppressive properties that have been described extensively in a previously reported review (28).

## Recognition of *Aspergillus fumigatus* by Bronchial Epithelial Cells

Schematically, the interaction between *Aspergillus fumigatus* and the BECs leads to three main types of cellular response: internalization, synthesis of cytokines/chemokines and release of bioactive molecules potentially active against *Aspergillus*. Specific interactions between *A. fumigatus* and BECs require close contact between the fungus and the cell-surface ligands. Fungal cell-wall polysaccharides and, to a lesser extent, some proteins or the genetic material (ADN or ARN) act as pathogen-associated molecular patterns (PAMPs). They are sensed through pathogen recognition receptors (PRRs), several of which but not all, have been identified in BECs (5) (Table 1).

**Table 1:** Pattern recognition receptors (PRRs) and pathogen-associated molecular patterns (PAMPs) involved in the recognition of *Aspergillus fumigatus* by bronchial epithelial cells and the consequences of their activation.

**PRRs**	***A. fumigatus* PAMPs**	**Cellular study model**	**Role in Anti-*Aspergillus* immunity on BECs**	**References**
Dectin-1	β-D-Glucan	HBE cells	Internalization Pro-inflammatory cytokines and chemokines release Inflammasome activation ROS generation	(29)
TLR2	β-D-Glucan	HBE cells	IL-6 and IL-8 release Increase in Dectin-1 expression	(29)
MR	Mannose-rich polysaccharides		Not elicited	
Unknown	FleA (conidia)	BEAS-2B cells	IL-8 synthesis Inhibition of *Aspergillus fumigatus* germination of extracellular conidia Binding of conidia to mucins and to macrophages	(30–32)
TLR3	dsRNA (resting conidia)	Primary human BECs	Release of inflammatory mediators, interferon (IFN)-β and IFN-γ-inducible protein (IP)-10	(33)
TLR4	Unknown		Not elicited	(34, 35)
TLR9	Hypomethylated DNA		Not elicited	(34, 35)
Pentraxin 3	Galactomannan		Not elicited	(36)

*BECs, Bronchial epithelial cells; IL, Interleukin; dsRNA, double stranded RNA; ROS, Reactive oxygen species; HBE cells, Papilloma virus–immortalized bronchial epithelial cell line*.

**Figure 1:**
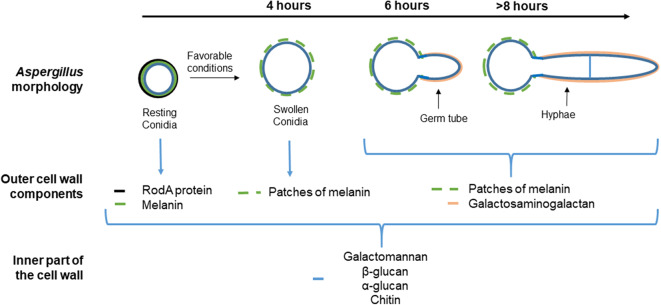
Different morphological stages and compositions of the cell wall during the vegetative cycle of *Aspergillus fumigatus*. The cell wall of the resting conidia consists of (the most external part to the inner) (i) the rodlet layer (black), composed of the hydrophobin RodA, (ii) the melanin layer below (green), and (iii) polysaccharides (blue). Under favorable conditions, the resting conidia begin to swell after 4 h. The rodlet layer is lost by proteolytic degradation and conidium swelling is due to an increase in the internal osmotic pressure. The melanin layer is then disorganized and the inner layer of the swollen conidium forms the mycelium cell wall. After 8 h, hyphae are apparent. Then, an extracellular matrix mainly composed of galactosaminogalactan (GAG) (pink) covers up the hyphae. Specific and universal components of each morphotype are shown below each image.

*A. fumigatus* cell wall encompasses an inner and outer layer whose composition varies along with the fungus' life cycle (Figure 1). The outer part of the conidial surface is composed of hydrophobic RodA proteins that conceal an underlying fungal pigment, dihydroxynaphthalene (DHN) melanin (37). This outer layer plays a key role in conidial dispersion, their protection against external stress factors such as desiccation, physical damage, drugs, and UV radiation. They also mask the epitopes present in the underlying layer by inhibiting their recognition by the host's innate immune system (38–40). Whereas, dormant conidia are described as immunologically inert, the FleA lectin, a fucose-binding lectin expressed on their surface (30, 41), has been shown to mediate their binding both to the airway mucins produced by the epithelial cells and also to macrophages (31). Moreover, stimulation of BEAS-2B cells by FleA has been reported to lead to an increase in interleukin (IL)-8 synthesis and contributed to the inflammatory response (30). Thus, FleA acts as a PAMP-like molecule whose cellular ligands remain to be identified.

Under favorable conditions, the dormant conidia become metabolically active and ensue an increased intracellular osmotic pressure followed by water uptake and isodiametric growth (42). The resulting swollen conidia lose their rodlet layer by proteolytic degradation, and consequently, their hydrophobicity. This phenomenon is associated with a rupture of the melanin layer by a still-unknown mechanism (43). Then, the swollen conidia grow in a polarized way that leads to the formation of a germ tube. At this stage, the melanin layer is disrupted but the inner layer of the cell wall remains unchanged and participates in hyphal formation. The hyphae are mainly composed of galactosaminogalactan (GAG) that allows adhesion of the filaments to various biotic or abiotic surfaces (44, 45). Recent studies have also shown that GAG, expressed during conidial germination, exhibits a possible anti-inflammatory effect. Indeed, GAG induces the release of the IL-1 receptor antagonist (IL-1Ra), a potent anti-inflammatory cytokine that blocks IL-1 signaling (46), by macrophages and neutrophils. Furthermore, in a mouse model of aspergillosis, treatment by GAG before and during intranasally induced *A. fumigatus* infection inhibits neutrophil infiltration in the lung at the site of infection (6, 44, 46).

Regardless of the *A. fumigatus* morphotype, the inner layer, the so-called fibrillary core, is continuously composed of branched β-(1, 3)-glucan/β-(1, 4)-glucan, chitin, galactomannan, and α-(1, 3)-glucan (43). A few studies have shown that, as already demonstrated for macrophages and digestive epithelial cells (47), Dectin-1 is a major but not the unique receptor for β-glucan on a papilloma virus-immortalized BEC cell line (HBE cells) (29). Dectin-1 is a transmembrane receptor and member of the C-type lectin receptor family (11). The role of Dectin-1 in the immune response against *A. fumigatus* has been highlighted in different *in vivo* studies. For example, immunocompetent mice lacking Dectin-1 are more sensitive to intratracheal challenge with *A. fumigatus* than control mice (48). In humans, mutations in Dectin-1 are associated with increased susceptibility to IPA (49). It has also been shown that immunocompromised mice, transfected to upregulate Dectin-1 expression in airway epithelial cells, have a lower fungal burden, an increase in the recruitment of neutrophils into the lungs and a greater survival rate in response to intratracheal injection of *A. fumigatus* conidia compared to the controls (50). After ligation of β-glucan to Dectin-1, two distinct signaling pathways are activated through the spleen tyrosine kinase (SYK)-caspase recruitment domain-containing protein 9 (CARD9) or through RAF-1 (51). These pathways act synergistically to induce nuclear factor-κB activation and pro-inflammatory gene expression (52). The SYK–CARD9 pathway also activates the NOD-, LRR-, and pyrin domain-containing 3 (NLRP3) inflammasome, which results in the proteolytic activation of the pro-inflammatory cytokines IL-1β and IL-18 by caspase 1. The role of *A. fumigatus* antigens in this activation cascade has been demonstrated by Jeong et al. They showed that sensitized mice intratracheally challenged with *A. fumigatus* crude antigens displayed an increased in immunofluorescence intensities of NLRP3 and caspase-1 in lung tissue, particularly in epithelial cell layers, leading to an increase in IL1-β concentration in the lung tissue (53). Similar results were obtained by using an *in vitro* model utilizing primary human BECs stimulated with the same *A. fumigatus* antigens (53). The mannose receptor (MR) is another C-type lectin receptor involved in fungal, and notably *Aspergillus* conidia, recognition and is expressed by 16HBE cells and primary BECs (54). The MR recognizes carbohydrates rich in mannose typically produced by many microorganisms including fungi (55) but its immune-specific role against *A. fumigatus* in association with BECs has not been investigated.

Toll-like receptors (TLRs) are another family of conserved PRRs (56). So far 10 different TLRs have been identified and described. TLR2, TLR4, and TLR9 are the main molecules involved in sensing fungal components (11, 57, 58). These receptors possess extracellular leucine-rich repeat ligand-binding domains and a conserved intracellular toll/IL-1R (TIR) signaling domain that induces specific signaling cascades through intracellular TIR containing adaptors such as MyD88. Interestingly, the expression of the 10 TLRs has been detected by reverse transcription–quantitative real-time polymerase chain reaction in two independent studies using human primary BECs (34, 35). Using a silencing method, it has been demonstrated that TLR2, in a heterodimer form with TLR1 or TLR6, is required for the expression of Dectin-1 by HBE cells in response to *A. fumigatus* infection (29). Moreover, blocking TLR2 with antibodies results in the inhibition of the release of IL-6 and IL-8 by BEAS-2B cells infected with *A. fumigatus* hyphal fragments (59). Certain polymorphisms in TLR4, receptor for the bacterial lipopolysaccharide, are clearly associated with increased susceptibility to invasive aspergillosis (60). TLR4 expressed by mouse macrophages, recognizes *A. fumigatus* conidia and hyphae and induces the release of pro-inflammatory molecules. However, after blocking TLR4 with a specific monoclonal antibody, Øya et al. failed to detect any significant change in the levels of IL-6 and IL-8 released by BEAS-2B cells infected with X-ray-treated hyphal fragments of *A. fumigatus* (59). Hypomethylated DNA, the natural ligand of TLR9, has been extracted in *A. fumigatus* hyphae (61). In murine model of invasive aspergillosis, Leiva-Juarez et al. revealed that therapeutic stimulation of lung epithelial defenses by inhalation of a synergistic combination of TLR 2/6 and TLR9 agonists robustly protects against the development of *A. fumigatus* lung infection despite the profound immune dysfunction (62). TLR9 is expressed by BECs (16HBE cells) (63) but its role in response to *Aspergillus* challenge remains to be investigated in the context of these cells. TLR3 is localized onto endosomal membranes and recognizes double-stranded (ds)RNA. It is primarily involved in the recognition of viruses but has also been implicated in the recognition of *A. fumigatus* dsRNA of resting conidia (33). In this study, the infection by *Aspergillus* of human primary BECs cultivated either in submerged or in ALI culture system, induced the release of inflammatory mediators, notably interferon-β and interferon-γ-inducible protein-10, through TLR3 signaling. Interestingly, this induction is internalization dependent, as demonstrated by the use of an actin-polymerization inhibitor and observed only with killed resting conidia (heat- or UV light-inactivated).

Finally, pentraxin 3, a soluble PRR, plays a key role in the recognition, uptake, and killing of *Aspergillus* conidia by macrophages and dendritic cells through binding to galactomannan (64). This molecule can act as an opsonizing factor for activating the complement system and subsequent phagocytosis by macrophages (65). Pentraxin 3 is secreted by both human primary bronchial and BEAS-2B cells but, again, its precise role in these cell types has yet to be determined (36).

## Internalization of *Aspergillus fumigatus* Conidia by Bronchial Epithelial Cells

**Figure 2:**
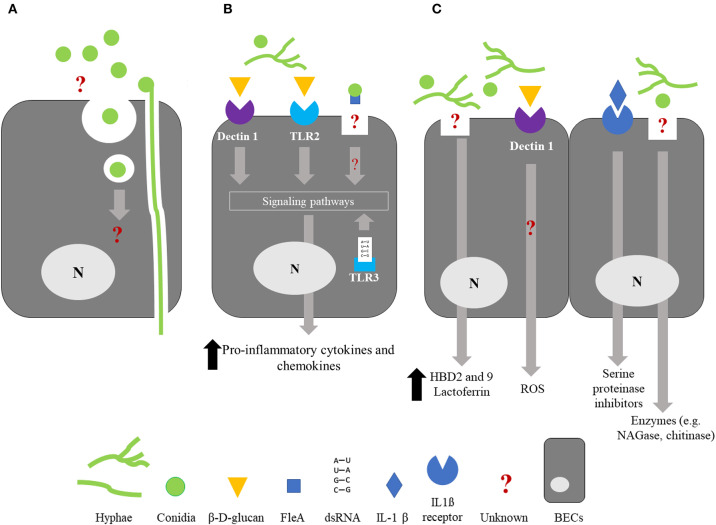
Summary of the interactions between bronchial epithelial cells and *Aspergillus fumigatus*. For clarity, it should be noted that the schematized epithelial cells are not polarized. **(A)** Internalization; BECs are able to internalized *A. fumigatus* conidia but the molecular mechanisms involved in this phenomenon and the fate of the internalized conidia are still unknown. An alternative way of penetration of the hyphae through an actin channel within the cell has been proposed. **(B)** Cytokine/chemokine release; Recognition of β-D-glucan by BECs through the membrane receptors Dectin-1 and TLR2, and recognition of *A. fumigatus* dsRNA by the endosomal receptor TLR3 result in the activation of intracellular signaling pathways and a significant increase in the release of proinflammatory cytokines and chemokines, leading to phagocyte recruitment. FleA lectin, expressed on the surface of *A. fumigatus* conidia, induces IL-8 synthesis by BECs and binding of the conidia to airway mucins. Recognition of FleA by BEC induces the inhibition of conidium germination. **(C)** Bioactive molecules potentially active against *A. fumigatus*; BECs synthetize antimicrobials molecules such as defensins HBD2 and HBD9, lactoferrin, reactive oxygen species (ROS), SLPI, ESI, and chitinase in response to *A. fumigatus* infection or inflammatory challenge. Until now, an antifungal activity has only been described for lactoferrin, ROS, SLPI, ESI, and chitinase. BECs, Bronchial epithelial cells; SLPI, Secretory leukocyte proteinase inhibitor; ESI, Elastase-specific inhibitor; NAGase, N-Acetyl-β-d-glucosaminidase; ROS, Reactive oxygen species; N, nucleus.

The alveolar epithelial cell line A549 has been extensively used to study conidia internalization by non-professional phagocytic cells (66–68). Wasylnka and Moore described that internalized conidia fused with lysosomes and colocalized with lysosomal protein (Lysosomal-associated membrane protein 1 and CD63). Nonetheless, a significant percentage of internalized conidia persist and germinate in A549 epithelial cells. Little is known about the internalization by BECs and conflicting results have been published (Figure 2A). First, Paris et al., showed by microscopic observations that rabbit tracheal epithelial cells were able to internalize *A. fumigatus* conidia after 6 h of incubation and that conidia were enclosed in membrane-bound vacuoles (69). Then, other studies reported that the conidia of *A. fumigatus* were taken up *in vitro* by a human bronchial epithelial cell line cultured in monolayers (70, 71). Indeed, Clark et al. observed the internalization of 10 to 20% of the conidia in contact with BEAS-2B cells at 6 and 9 h after challenge. In comparison, 70% of conidia in contact with macrophages were internalized after 1 h incubation (71). A 41% internalization rate has also been reported with 16HBE cells after 6 h incubation (70). Overall, these results contrast with those from other studies that have considered the internalization by BECs a very minor phenomenon. By using a model of primary BECs grown in ALI culture system, Toor et al. showed that only 1% of the bound conidia were internalized 6 h after exposure (72). Similarly, Fernandes et al. were unable to demonstrate any case of internalization using primary BECs cultured in ALI (73). However, these authors described the formation, within the cell, of an actin tunnel not altering the viability of the penetrated cells (Figure 2A). The authors suggested that this event could explain the penetration of the hyphae into the underlying parenchyma leading to the development of invasive infection in immunocompromised patients (73). It is noteworthy that the internalization of conidia by BECs was not seen 18 h after an intratracheal challenge in an immunosuppressed mouse model (74). The molecular mechanism(s) involved in the internalization of *Aspergillus* conidia by BECs remains incompletely understood. Adhesion of conidia onto BECs induces actin polymerization (75), a phenomenon dependent on the activity of the human actin reorganization complex 2 and 3, regulated by Wiskott-Aldrich syndrome protein-interacting proteins (76). Using the BEAS-2B cell line, Clark et al. identified 7 host markers—caveolin, flotillin-2, RAB5C, RAB8B, RAB7A, 2xFYVE, and FAPP1—that consistently localized around the internalized conidia (71).

Even if the internalization of *Aspergillus fumigatus* conidia by BECs is a rare event, however, it could represent a starting point of invasive forms in immunosuppressed patients. The fate of the internalized conidia is still unknown and one can postulate that some of them remain quiescent within the cells until they reactivate, thanks to iatrogenic immunosuppression (77). This could explain the breakthrough of some invasive aspergillosis cases occurring in immunocompromised patients otherwise protected from contamination from the ambient air using a high-efficiency particulate air filter chamber (78).

## Synthesis and Release of Bioactive Molecules by Bronchial Epithelial Cells Infected With *Aspergillus fumigatus*

### Cytokine/Chemokine Synthesis

One of the most studied consequences of PAMP/PRR interaction is the induction of cytokine/chemokine synthesis. Several studies have shown that infection of BECs with *Aspergillus fumigatus* leads to the release of pro-inflammatory cytokines, mostly IL-6, IL-8, or tumor necrosis factor-α provided that the infection time is more than 6 h and allows conidia germination (79–81) (Figure 2B). Hence, after 6 h of incubation, expression (mRNA) of not only tumor necrosis factor-α and IL-8 but also granulocyte macrophage-colony stimulating factor (GM-CSF) in HBE cells exposed to *A. fumigatus* conidia, is significantly increased by 8 to 14 times in regards to non-infected cells (29). Similarly, BEAS-2B cells release an increased amount of IL-8, 8 h after having been infected with *A. fumigatus* conidia, at a time corresponding to the hyphal formation (79) (Figure 1). In contrast, IL-8 synthesis was not triggered 15 h post-infection in BEAS-2B cells infected with a mutant strain of *A. fumigatus* unable to germinate (79). Considering the differences in the cell-wall content between the conidial and hyphal stages, these observations strongly suggest a role for some parietal molecules in masking resting conidia, avoiding their recognition by the BECs. RodA hydrophobin, whose masking role toward immune cells has been mentioned above, could be involved but, to the best of our knowledge, this hiding role toward BECs has never been checked.

Inflammatory mediators act synergistically to establish an organized and regulated host response against *Aspergillus fumigatus*. For example, IL-8, also known as CXCL-8, is a chemokine exhibiting a pleiotropic effect on neutrophils: strong chemotactic influence, degranulation of lysosomes with release of enzymes within the phagosome, production of reactive oxygen species (ROS), and increased expression of adhesion molecules (82). GM-CSF acts both as a hematopoietic growth factor favoring the proliferation and differentiation of myeloid cells into mature cells such as neutrophils and macrophages, and as an enhancer of the antimicrobial functions of those cells. Very recently, our group demonstrated that the inflammatory response of BEAS-2B cells and human primary BECs against *A. fumigatus* could be reprogrammed after the first contact with a microbial ligand, in this case *Pseudomonas aeruginosa* flagellin (81). Pre-stimulation with this TLR5 ligand led to a significantly enhanced release of two proinflammatory cytokines, IL-6 and IL-8, after an *A. fumigatus* challenge. This is comparable to the phenomenon called trained immunity or innate immune memory that has been largely studied using monocytes/macrophages (83–85).

### Molecules Potentially Active Against *Aspergillus fumigatus*

Antimicrobial peptides (AMPs) are cationic small-peptide chains that exhibit antimicrobial activity against a variety of pathogens including fungi (86). Although membrane permeabilization is the main mechanism of action of AMPs against pathogens, additional mechanisms have been described including inhibition of macromolecular synthesis (87). Under basal conditions, BECs release a number of AMPs or proteins, some of which exhibit potential antifungal activity (Figure 2C).

The defensin family, divided into three classes (α-, β-, and θ-defensins), includes broad-spectrum antimicrobial peptides that are evolutionarily conserved across the living world (88). Human β-defensins are a characteristic of epithelial tissues and present a constitutive expression in primary human BECs (89). Human β-defensin 2 and human β-defensin 9 are reported to be highly expressed by BECs (HBE and 16HBE cells) exposed to different morphotypes of *A. fumigatus* (29, 90). Lactoferrin is a protein synthesized and released by 16HBE cells (91). Interestingly, according to Lupetti et al., a synthetic peptide based on the human lactoferrin sequence but containing only the first cationic domain is one of the most potent antimicrobial peptides against *A. fumigatus* hyphae and conidia *in vitro* (92). Lactoferrin can also act by reducing the toxic effect on host cells (cytotoxicity, oxidation level, and DNA damage) of aflatoxin, a mycotoxin synthesized by *Aspergillus* (93). Secretory leukocyte proteinase inhibitor (SLPI), also called anti-leukoprotease or mucus proteinase inhibitor, and elastase-specific inhibitor (or elafin and trappin-2) are two serine proteinase inhibitors constitutively secreted from the airway epithelium (94). Both are secreted by BECs in response to pro-inflammatory cytokines such as IL-1β (95, 96). Different biological functions have been reported for these molecules: protection of the lungs against the damage induced by neutrophil serine proteases and also antimicrobial activity notably against fungi like *A. fumigatus* (97, 98). However, these proteins are not considered as AMPs because their size, 11.7 and 9.9 kDa for SPLI and elastase-specific inhibitor respectively, are too large to be classified as peptides.

Interestingly, BEAS-2B and HBE cells are also able to produce ROS (29, 99). In their study on BECs challenged with *A. fumigatus*, Sun et al. showed that conidia induced ROS generation in a Dectin-1-dependent manner after 6 h of infection (29). ROS are known to be produced by neutrophils thanks to nicotinamide adenine dinucleotide phosphate oxidase produced in response to germinating *A. fumigatus* challenge (100, 101). They are released from granules either into phagosomes or into the extracellular environment, inducing damage to *Aspergillus* (6). But until now, the precise role of ROS produced by BECs in antifungal activity remains undetermined.

The study of the secretome of BEAS-2B cells infected with *A. fumigatus* also gave more insight into the role of some molecules that may possibly act against the fungal infection (102). Among the most significant results, Fekkar et al. found the release of lysosomal enzymes such as N-Acetyl-β-d-glucosaminidase, cathepsin B, and cathepsin D. N-Acetyl-β-d-glucosaminidase is responsible for the hydrolysis of glycosidic bonds. Cathepsin B and D, members of the lysosomal cysteine protease family, are known to acidify the phagosome of macrophages but their role in BECs has not been determined yet. Chitinase, a member of N-Acetyl-β-d-glucosaminidase, degrades the major fungal-wall component chitin and as such could play a role in the control of *Aspergillus* infection (103, 104).

At this time, not all the active molecules may have been described. Recently, Richard et al. reported that BEAS-2B cells prevent the germination of conidia without internalization but failed to demonstrate the role of any soluble compound present in the supernatants of infected cells (32). This antifungal activity of BECs is fungistatic and occurs via a mechanism that is phosphoinositide 3-kinase dependent. The same kind of observation was made by Clark et al. who found that after infecting BEAS-2B cells with *A. fumigatus* conidia, a large subset of conidia is rendered metabolically inactive, as measured with the metabolic marker FUN-1, while not being internalized by the cells (71).

## Conclusion

The evidence of BECs playing an important role in the innate immunity-based defense mechanism against *Aspergillus fumigatus* is now growing. Recent studies have been able to demonstrate that, in addition to the production of mucus and ciliary beating that allow the clearance of the conidia, BECs are directly involved in an immune response against *A. fumigatus* through the recognition of fungal cell-wall components, mainly polysaccharides, by cellular ligands such as Dectin-1 or TLRs. There is a consensus for a pro-inflammatory response by BECs stimulated by *A. fumigatus*. However, additional studies are needed to better decipher BECs response. Studies focused on the internalization of the conidia by BECs reported divergent results according to the study model. This warrants further studies to clarify this point such as the investigation of the fate of the internalized conidia that could play a role in the future development of invasive aspergillosis. Different types of AMPs are also produced by BECs, some of them being active directly or indirectly against *Aspergillus*, making these molecules appealing for new therapeutic approaches. Overall, the bronchial epithelium appears as a suitable target for novel therapeutic strategies aiming to restore barrier integrity and to enhance defenses against inhaled pathogens.

## Author Contributions

JB, VB, and CH: drafting of the manuscript. JB, VB, CH, LG, JG, MR, and HC: revision of the manuscript.

## Conflict of Interest

The authors declare that the research was conducted in the absence of any commercial or financial relationships that could be construed as a potential conflict of interest.

## Abbreviations

BECs: Bronchial epithelial cells
IPA: Invasive pulmonary aspergillosis
CF: Cystic fibrosis
PCD: Primary ciliary dyskinesia
ABPA: Allergic bronchopulmonary aspergillosis
ALI: Air-liquid interface
DHN: Dihydroxynaphthalene
HBE cells: papilloma virus–immortalized bronchial epithelial cell line
PAMPs: Pathogen-associated molecular patterns
PRRs: Pathogen recognition receptors
IL-1Ra: Interleukin-1 receptor antagonist
NLRP3: NOD-, LRR-, and pyrin domain-containing 3
ROS: Reactive oxygen species
IL: Interleukin
AMPs: Antimicrobial peptides
hBD: Human β-defensins
NAGase: N-Acetyl-β-d-glucosaminidase.
